# MAPPER: a search engine for the computational identification of putative transcription factor binding sites in multiple genomes

**DOI:** 10.1186/1471-2105-6-79

**Published:** 2005-03-30

**Authors:** Voichita D Marinescu, Isaac S Kohane, Alberto Riva

**Affiliations:** 1 Children's Hospital Informatics Program, Children's Hospital Boston, Harvard Medical School,300 Longwood Avenue, Boston, MA 02115, USA

## Abstract

**Background:**

*Cis*-regulatory modules are combinations of regulatory elements occurring in close proximity to each other that control the spatial and temporal expression of genes. The ability to identify them in a genome-wide manner depends on the availability of accurate models and of search methods able to detect putative regulatory elements with enhanced sensitivity and specificity.

**Results:**

We describe the implementation of a search method for putative transcription factor binding sites (TFBSs) based on hidden Markov models built from alignments of known sites. We built 1,079 models of TFBSs using experimentally determined sequence alignments of sites provided by the TRANSFAC and JASPAR databases and used them to scan sequences of the human, mouse, fly, worm and yeast genomes. In several cases tested the method identified correctly experimentally characterized sites, with better specificity and sensitivity than other similar computational methods. Moreover, a large-scale comparison using synthetic data showed that in the majority of cases our method performed significantly better than a nucleotide weight matrix-based method.

**Conclusion:**

The search engine, available at , allows the identification, visualization and selection of putative TFBSs occurring in the promoter or other regions of a gene from the human, mouse, fly, worm and yeast genomes. In addition it allows the user to upload a sequence to query and to build a model by supplying a multiple sequence alignment of binding sites for a transcription factor of interest. Due to its extensive database of models, powerful search engine and flexible interface, MAPPER represents an effective resource for the large-scale computational analysis of transcriptional regulation.

## Background

Identifying the combinatorial logic of transcriptional regulation is key for understanding the mechanisms of development, cell commitment and differentiation and the way in which external and internal signals are converted into specific patterns of gene expression. Transcriptional regulation is accomplished by the coordinated activity of specific regulatory proteins that recognize and bind regulatory elements – short DNA motifs located in the untranscribed regions of the genes [1]. Regulatory elements such as TFBSs, enhancers, and silencers, are commonly located in the promoter region of genes, while others, such as splicing control elements, may be located within the introns or exons of a gene. As more sequence and expression data have become available, the task of understanding gene regulation has come to rely on a combination of experimental and computational approaches. Among the many bioinformatics approaches aimed at understanding the role of regulatory elements in transcriptional control, several research themes have emerged [2-5]. They include search algorithms that extract putative regulatory elements [6-15], search engines for their retrieval [16-19] and databases of experimentally characterized or computationally derived regulatory elements [20-24]. Moreover, combinations of regulatory elements that occur in close proximity to each other form *cis*-regulatory modules that control gene expression. Their presence suggests the existence of a combinatorial code for transcriptional regulation [25], with ample effort being devoted to developing algorithms for its elucidation [26-34].

In sequences of orthologous genes, regulatory elements that have a functional role are often conserved throughout evolution by selective pressure. This led to the development of many algorithms that use 'phylogenetic footprinting' – a method for inferring regulatory elements based upon sequence conservation of orthologous genes [35-45]. However, recent evidence [46] shows that functional elements are not necessarily located in conserved regions; this requires the development of computational methods to detect binding sites with high specificity without relying primarily on sequence conservation data.

One of the most common strategies for identifying putative TFBSs in DNA sequences relies on matching a general pattern abstracted from sequences of experimentally characterized binding sites and expressed in the form of a probability weight matrix that describes the probability distribution of the four possible nucleotides at each location [47]. Several programs, such as Patch, Match, MatInspector and TESS, rely on the nucleotide weight matrices (NWMs) of TRANSFAC – a large and frequently updated database that contains information on the transcription factors (TFs) and their binding sites in target genes [20]. The assumption underlying the construction and use of NWMs is that each nucleotide contributes independently to the binding site consensus and that the contribution of the nucleotides to the site is additive [48]. This assumption was tested experimentally in the case of binding sites for two transcription factors – the Mnt repressor protein and the mouse EGR1 protein. Nucleotides at positions 16 and 17 in the Mnt repressor protein binding site [49] and the central nucleotide triplet in the mouse EGR1 binding site [50] were systematically mutated to all possible combinations and the binding affinity of the respective TFs (or its mutants) for these sites was determined. The results pointed out that the assumption of independence of nucleotides within a site is not entirely accurate, but that although NWMs do not capture the dependencies between nucleotides within a site they represent a good enough approximation for modeling it[48,49]. Nevertheless, it is generally recognized that using NWMs to identify putative TFBSs often leads to the retrieval a very high number of false positives [47].

In this work, we rely instead on Hidden Markov Models (HMMs) as a more accurate probabilistic method to model the sequence of nucleotides within a binding site that in addition to abstracting the probability distribution of the nucleotides at each site can also model insertion or deletions and retrieve fragment matches to the model in the search procedure [51]. HMMs are statistical models able to represent stochastic sequences of symbols and can be used to generate sequences that conform to a given model, or to determine the likelihood that a given sequence was generated by that model. HMM techniques have become the basis of many bioinformatics applications for recognizing conserved domains in amino acid sequences or gene features in DNA sequences [51,52]. Several publicly available implementations such as HMMER [51], SAM [53] and Meta-MEME [54] build HMMs based on multiple sequence alignments and, among other functions, search input sequences for matching domains. The HMMER package was used to generate the large collection of annotated protein domains of the Pfam database [55] and consists of several modules for which the source code is available, well commented and easily modifiable. HMMs were previously considered for modeling and searching for TFBSs. The reports so far were either theoretical in nature [50,56], where not extended to genome-wide searches [57], or focused only on a small number of transcription factors [58-60]. A HMM for CREB binding sites was used to scan upstream sequences of 10 kb in length from the human and mouse genomes [58], while a Markov model for the hepatocyte nuclear factor 4 (HNF4) was used to scan sequences between positions -500 to +100 relative to the transcription start site of confirmed genes in the human genome [59]. Recently, a method for identifying nuclear hormone receptor bindings site was developed based on the use of classification HMMs [61]. However, to date no study used HMMs of multiple TFBSs in a large-scale search across three or more genomes.

The work described in this paper focuses on developing methods to generate accurate and complete information on putative TFBSs in genes across multiple genomes (*H. sapiens*, *M. musculus*, *D. melanogaster*, *C. elegans *and *S. cerevisiae *in the initial implementation). Our methodology relies on combining the information on experimentally determined binding sites contained in curated databases such as TRANSFAC and JASPAR with the pattern matching power of HMMs. As TRANSFAC and JASPAR provide manually curated representative nucleotide sequences of binding sites for most TFs included in the databases, we leveraged this information to recreate the alignments used to calculate the NWMs, and we used them as input to HMMER instead. We thus generated a library of 1,079 HMM profiles containing one model for each TRANSFAC matrix or factor entry (see below) or JASPAR matrix for which alignments of binding sites were available. The performance of selected models was evaluated by their ability to retrieve experimentally characterized binding sites, and the sensitivity and specificity of the method were assessed in a large-scale comparison with a NWM-based method using synthetic data. A flexible interface was implemented (and is publicly available at ) that allows the user to search a sequence in FastA format or a gene and its orthologs across five genomes against the library of 1,079 HMM models or against a model built by the user starting with a multiple sequence alignment of binding sites. Although the tools developed for this work were used to find putative TFBSs, they are directly applicable for identifying other types of regulatory elements for which sequence alignments are available.

## Results

### The MAPPER HMM library

**Figure 1:**
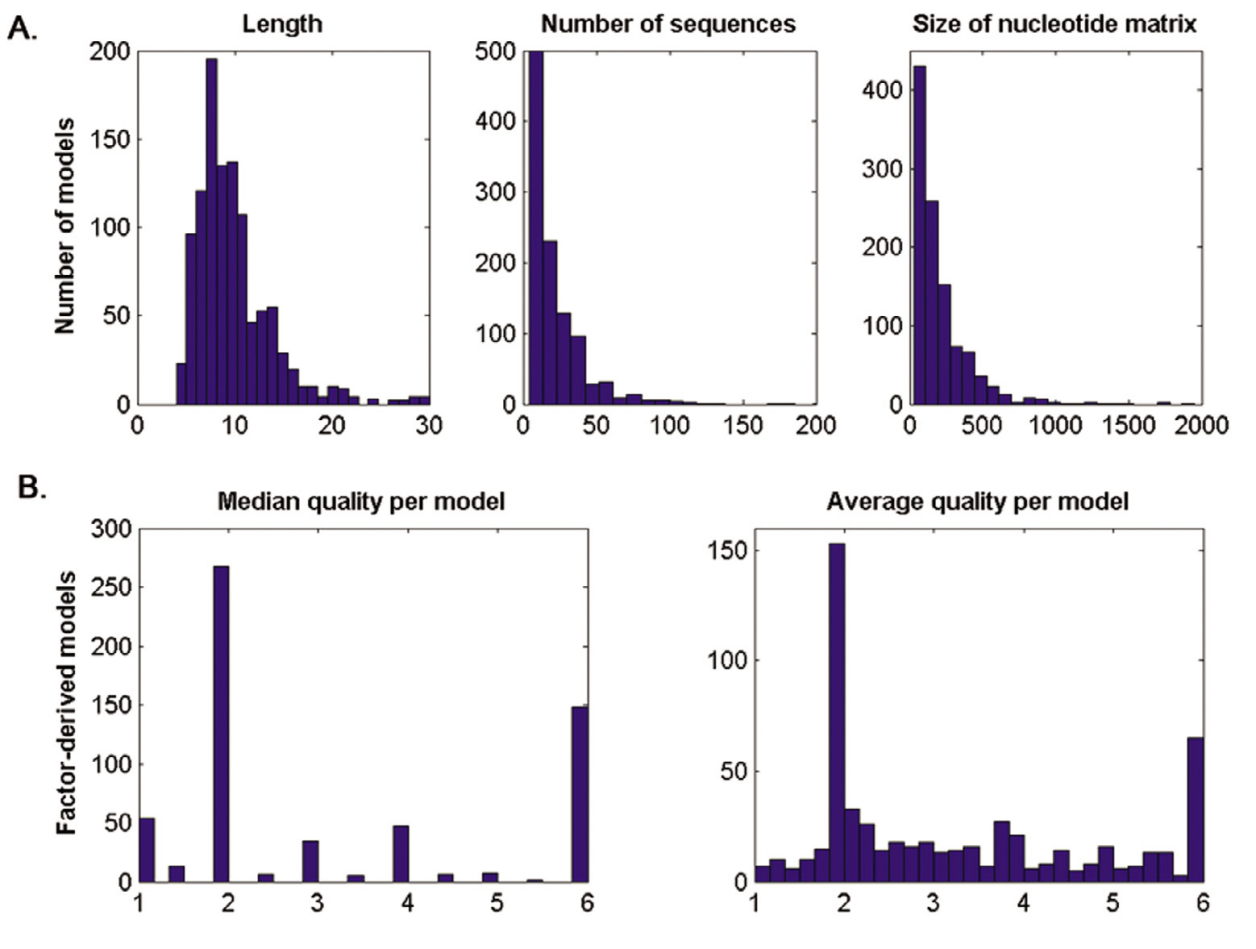
**Quality measures for the alignments retrieved**. A. Distribution of the parameters characterizing the model (length, number of sequences and the size of the nucleotide matrix used to train the model). B. Distribution of the median and average quality of the nucleotide sequences used to build the alignments for the TRANSFAC factor-derived models. The quality variable is categorical and represents "1 – functionally confirmed factor binding site; 2 – binding of pure protein purified or recombinant, 3 – immunologically characterized binding activity of a cellular extract, 4 – binding activity characterized via a known binding sequence, 5 – binding of uncharacterized extract protein to a *bona fide *element, 6 – no quality assigned" (cf. TRANSFAC documentation).

HMMs were built using multiple sequence alignments of binding sites compiled from the TRANSFAC [20] and JASPAR [21] databases. TRANSFAC provides two sources of information regarding the binding sites for TFs: nucleotide sequences of binding sites referenced in the description of the TRANSFAC matrices that were optimally aligned and used to derive NWMs (designated below as matrix-derived alignments and catalogued with accession numbers starting with "M"), and nucleotide sequences of binding sites referenced as part of the description of the TFs – also referred to as "factors", used to extract alignments designated below as factor-derived alignments and catalogued with accession numbers starting with "T". By parsing the TRANSFAC flat files (see Methods for details) we obtained 402 alignments corresponding to matrices and 588 alignments corresponding to factor entries. In addition, 89 alignments were obtained from data downloaded from the JASPAR database. Thus, the total number of alignments used to build HMMs was equal to 1,079. Figure 1A shows the distribution of the length of the models and of the number of sequences and size of the nucleotide matrix used to train them. The models have an average length of 10 nucleotides and were trained on an average of 22 sequences. TRANSFAC assigns a quality value to the sites used to build the factor-derived models, based on the existing biological evidence of the binding (see Figure 1B legend for details). The distribution of the median and average quality of the sites used for the factor-derived models suggests that the large majority contains high quality sites (categorical values smaller than 4).

The 1,079 models retrieved correspond to 888 transcription factors entries with distinct names in TRANSFAC and JASPAR. Table 1 in Additional File 1 lists the names of all TF entries in the two databases for which HMMs were built and the models that describe them. It is important to note that different databases (or even the same database) often use different names for the same TF or for isoforms of the same TF (e.g. p65, RelA; HNF-1, HNF-1alpha); nevertheless, for the purpose of this paper, entries with different names were considered as distinct.

While the matrix-derived models are generated by combining binding sites for homologous factors from multiple organisms, every factor-derived model and JASPAR model is derived from sites from a single organism. Our search engine does not place restrictions on the use of a model associated with a TF from one organism when searching a sequence from a different organism. The rationale for this is that ortholog TFs from different organisms usually show very high structural and functional conservation that extends to their binding site specificities. This allows the user to use all available models for a given TF when searching, and to evaluate *a posteriori *whether the resulting hits are significant.

### Evaluation of the method

Evaluating the sensitivity and specificity or our method compared to other commonly used ones is not straightforward, for a variety of reasons. First, in order to measure the false positive and false negative rates we would need to be able to reliably classify occurrences of the motif (also referred to as "hits") into "true" and "false" positive categories. This is obviously impossible by computational means, and too expensive and time consuming to pursue experimentally for a large number of transcription factors and binding sites. On the other hand, the experimental data sets that make available genome-wide positions of "true" hits pertain to a limited number of factors of interest and usually report a region for which binding was detected and not the precise locations and sequences of the binding sites [62-65]. For a limited number of well-characterized factors, collections of binding sites were compiled from the literature (see below) but the total number of such sites is still too small to enable a statistically significant comparison. As such sites usually come from promoter regions that are rich in regulatory sites, we were forced to use short flanking sequences to avoid including extraneous additional sites. The results of testing a method on such short sequences are not entirely predictive of its performance when long genomic regions are used as input, as in most experiments. Moreover, for all these datasets and even for other ones that contain binding data for multiple factors [66], no information is available about "false" hits (i.e. sites that match a consensus but are not functional). Although it is clear that only a large-scale biological validation of the predictions of this method can provide a definite estimation of its performance, such goal is beyond the scope of this paper.

Given these constraints, we performed three types of evaluations of our method. First, for three different factors we performed control runs to determine if our method is able to correctly identify a total of 17 experimentally characterized binding sites in 9 different genes with the exact sequence and at the positions reported in the literature. Secondly, for a collection of 89 experimentally characterized sites for six other transcription factors, we determined the number of sites retrieved and the percentage of false positives reported by our method and compared them with the results of four other methods: Match [19], Patser [9], LMM [67] and ScanACE [6]. Finally, we conducted a large-scale evaluation based on synthetic data for 491 models in our database in order to compare, using modified ROC curves, the sensitivity and specificity of our method with the ones of a NWM-based method (in this case Match).

#### Control runs

To obtain a preliminary evaluation of the performance of our method in detecting TFBSs we used control sequences consisting of promoter regions of genes in which binding sites for specific factors were determined experimentally and were shown to play a role in the regulation of the gene. We selected as controls HMM models for three transcription factors – p53, Su(H) and MEF-2 and used them to scan control sequences chosen so that they contain experimentally determined TFBSs for the factors whose nucleotide sequence were not included in the multiple sequence alignments used to train the models.

A HMM corresponding to p53 was built based on the alignment for matrix M00761 of TRANSFAC, and was used to scan promoter regions from the following genes: the human *14-3-3 sigma protein *gene for which two p53 binding sites, BDS-1 and BDS-2, were characterized [68], the mouse *cyclinG1 *gene that contains one p53 binding site [69], and the mouse *B99/Gtse1 *gene, encoding the G two S phase expressed protein 1 for which a p53-responsive element containing three p53 half-sites was reported [70]. With the exception of one half-site in the latter sequence, HMMER retrieved all the described p53 binding sites at the positions indicated in the literature, with significant scores and *E*-values (for the definitions of these parameters see the Methods section). Model M00234 for the *Drosophila *Su(H) (Suppressor of Hairless) was used to scan the following sequences: the promoter of *Drosophila him *[71] containing four Su(H) binding sites that were identified computationally and confirmed experimentally, the sequence of an enhancer containing three Su(H) sites located 3.5 kb upstream of the first exon in *Drosophila yan *[72] and the promoter sequence of the human *erbb-2 *gene containing one Su(H) binding site [73]. While the sites in *Drosophila him *where identified with positive scores, the binding sites in the last two sequences were reported with negative scores, pointing out that, as mentioned in the HMMER documentation [74], real matches can in some cases have negative scores (one of the three Su(H) sites in the *Drosophila yan *enhancer was missed). Model T00505 corresponding to human MEF-2 (myocyte-specific enhancer factor 2A) was used to search the promoter sequences from the following genes, each containing one well characterized MEF-2 site: *Drosophila Actin57B *gene [75], mouse *mef2c *gene [76] and human *c-jun *[77]. The characterized MEF-2 binding sites in these sequences were also retrieved by HMMER (with a negative score for *c-jun*). In total our method identified correctly 15 out of the total 17 binding sites (the complete list of hits can be accessed following the appropriate link from Additional File 1).

#### Small-scale evaluation

The purpose of this evaluation was to measure the performance of our method compared to other widely used tools for the computational detection of TFBSs, using a high-quality dataset of experimentally validated binding sites. The criteria we used to compare the TFBS detection methods in our experiment are the following: first, we required a method to be able to detect all the experimentally validated sites (*true positives*) in the input sequences, except for one at most. Next, we counted the number of hits not corresponding to true sites (*false positives*) having a score greater then the lowest-scoring true positive, and we expressed it as a percentage of the sum of the number of true positives and false positives. According to this definition, the false positives represent those hits that cannot be separated from the true ones on the basis of their score, since raising the score threshold to exclude them would cause the method to miss some true positives.

The dataset used for this evaluation contained 110 experimentally characterized binding sites for six factors: E2F, the estrogen receptor (ER), the glucocorticoid receptor (GR) and the hepatocyte nuclear factors 1, 3 and 4 (HNF-1, HNF-3 and HNF-4) previously compiled from the literature [78-80]. For each factor the dataset contained a 50 bp long sequence extracted from a given gene in which the binding site was always located in the same positions throughout the dataset (e.g. for all sequences in the E2F dataset the actual site was found between positions 20–31). The length of the sites was 11 for HNF-3, 12 for E2F, 13 for ER and HNF-1, 14 for GR, and 15 for HNF-4. Adopting this consistent format for the datasets allowed us to define a true positive as a hit that overlaps at least 60% of the sequence of the site and a false positive as any other hit retrieved in the sequence. Hits retrieved on both strands that overlapped more than 75% were considered equivalent and only the best scoring one was reported.

We included in this evaluation four other methods for TFBS detection: Match [19] and Patser [9] that scan a sequence using a supplied NWM, LMM (Local Markov Method) [67] that uses a *p*-value-based scoring measuring the similarity of the hit to the known binding sites for the factor and its contrast to the local genomic context, and ScanACE [6] that scans a sequence for matches for a given motif using a scoring method based on a maximum *a priori *log likelihood score. In order to conduct a fair evaluation for each factor we used as input either the HMM (for our method) or the corresponding NWM (for Match, Patser and LMM – see exceptions below) that were built on the same alignment. This alignment was also used directly as input for ScanACE. For three factors (ER, GR and HNF-1) the matrix used in the analysis was not found in the LMM matrix library, therefore we used the closest matrix available as judged by examining its consensus sequence. To preserve this correspondence we were in some cases forced to use a sub-optimal model in the HMMER run; in these cases we also included an alternative HMM model in the analysis (the best one available to HMMER for the given factor). We also filtered the collection of binding sites in order to eliminate the ones that appear in the training set (i.e., that were used in the alignments on which the matrices and all HMMs used were built), resulting in a dataset that contained a total of 89 sites. Information regarding the binding sites in the alternative matrices used for LMM is not available in TRANSFAC, so it is possible that in these cases the alternative matrix used overfits the dataset.

The results of the small-scale evaluation are presented in Table 1. The complete listing of the hits found (including their position, sequence and score) as well as the consensus of the models and matrices used is provided in the Additional File 2. Table 2 summarizes the results: for each method tested we report the overall percentage of true positives identified, and the lowest and highest percentage of false positives in each of the six datasets. In several cases, two of the methods tested (LMM and ScanACE) did not reach a sufficient number of true positives, so their performance is not directly comparable to the one of the other methods. Their performance in terms of false-positive hits suggests that they are too specific, and therefore prone to missing true sites. The other two programs that we tested against, Match and Patser, show a minimum false-positive percentage of 12% and 3% respectively. In contrast, HMMER reaches a minimum value of 0 false-positive hits, while detecting all (or almost all) true sites in all cases. The highest percentage of false positive hits ranges from 16.7% for HMMER (selecting the optimal model) to 43.7% for Patser. While on individual models other methods might perform better in particular cases, these results indicate that HMMER is powerful enough to detect the target binding sites in all the datasets tested, and that its sensitivity-specificity trade-off is consistently better than those of the other methods.

#### Large-scale evaluation

To add to the rigor of this analysis and perform it in a context closer to a real-life biological investigation, we resorted to a computational evaluation based on synthetic data generated similarly to the technique described by Barash et al. [81]. This allowed us to compare the proportion of false positive hits returned by our method and by a TFBS prediction program based on NWMs, in this case Match [19]. We applied the following procedure to the 491 HMMs in our database (corresponding to all TRANSFAC matrix-derived and JASPAR-derived models) for which a corresponding NWM built using the same multiple sequence alignment was available: we generated a random nucleotide sequence of a fixed length (50,000 bp) and we inserted in it, at random locations, 100 "synthetic" binding sites. These binding sites were not generated by sampling from the matrix nor from the HMM as that could have conferred an advantage to one of the two methods. Instead the sites were generated by sampling from the alignment with an algorithm designed to make the test as fair as possible for both methods by preserving both the dependencies between nucleotides in the sequences and the core matrix if it exists (see Methods for details). Before each hit was planted the random sequence was scanned to eliminate any other occurrence of its sequence that might have been present by chance. We defined our planted hits as the "true positives", while any other reported instance of the pattern was considered a "false positive". We then scanned the resulting nucleotide sequence with the Match program and with HMMER, we repeated the experiment 20 times for each model independently and we averaged the results to eliminate fluctuations due to randomization.

We measured the performance of the two methods using modified ROC curves – ROC_50 _curves [82] and compared the areas obtained with both methods by using a Bonferroni-corrected Wilcoxon signed rank test. Out of the original 491 models tested 105 were eliminated due to the fact that the average ROC_50 _area for either method was smaller than 0.25, leaving 386 filtered models on which the comparison was conducted. At a significance level of 0.05 and with a Bonferroni correction of 491, our method performed better than Match for 96% of the models for which the result was significant (71% of the filtered models), suggesting that it can provide a better trade-off between sensitivity and specificity that translates into being able to retrieve more true positives with fewer false positives. The results of this test are presented in Table 2 of Additional File 1. It should be noted that in many cases the length of the matrix used by Match differs from the length of the HMM even though the same alignments were used to construct both. This situation confers an advantage to Match because the planted hits have the same length as the alignment.

We then analyzed the results of these runs to determine, for each method and model, the percentage of true positive hits within the first *n *reported hits (the greater this percentage, the more sensitive the method is), and the amount of false positives expressed as a percentage that needs to be accepted in order to retrieve the first *m *true positive hits (the smaller this percentage, the more specific the method is); these tests are referred below to as the first and second TPP (true positive proportion) tests. The values used for *n *and *m *were 30, 50, 70 and 90. Tables 3 and 4 of Additional File 1 show these results for all 386 filtered models tested. While the ROC tests give an indication of the overall performance of the method, the TPP tests assess its performance in the "early" and "late" stages of the search, respectively. By the stringent criteria used for comparison (see Methods) in the first TPP test our method performs better for 78% of the cases for which a difference was noted suggesting that these models are better suited for retrieving a relatively small number of hits that are true positives (as for example when analyzing the promoter of a given gene) while reporting a minimal number of false positives. In the second TPP test our method performs better for 85% of the cases in which a di fference in performance between the two methods was noticed, suggesting that these models can retrieve a large number of hits while limiting the number of false positives, as for example in the case of a genome-wide search for putative TFBSs for a given factor. It should be noted that since each of the three tests measures a different aspect of the performance of the method, the list of models that perform better in each case might not entirely overlap.

### The MAPPER interface

**Figure 2:**
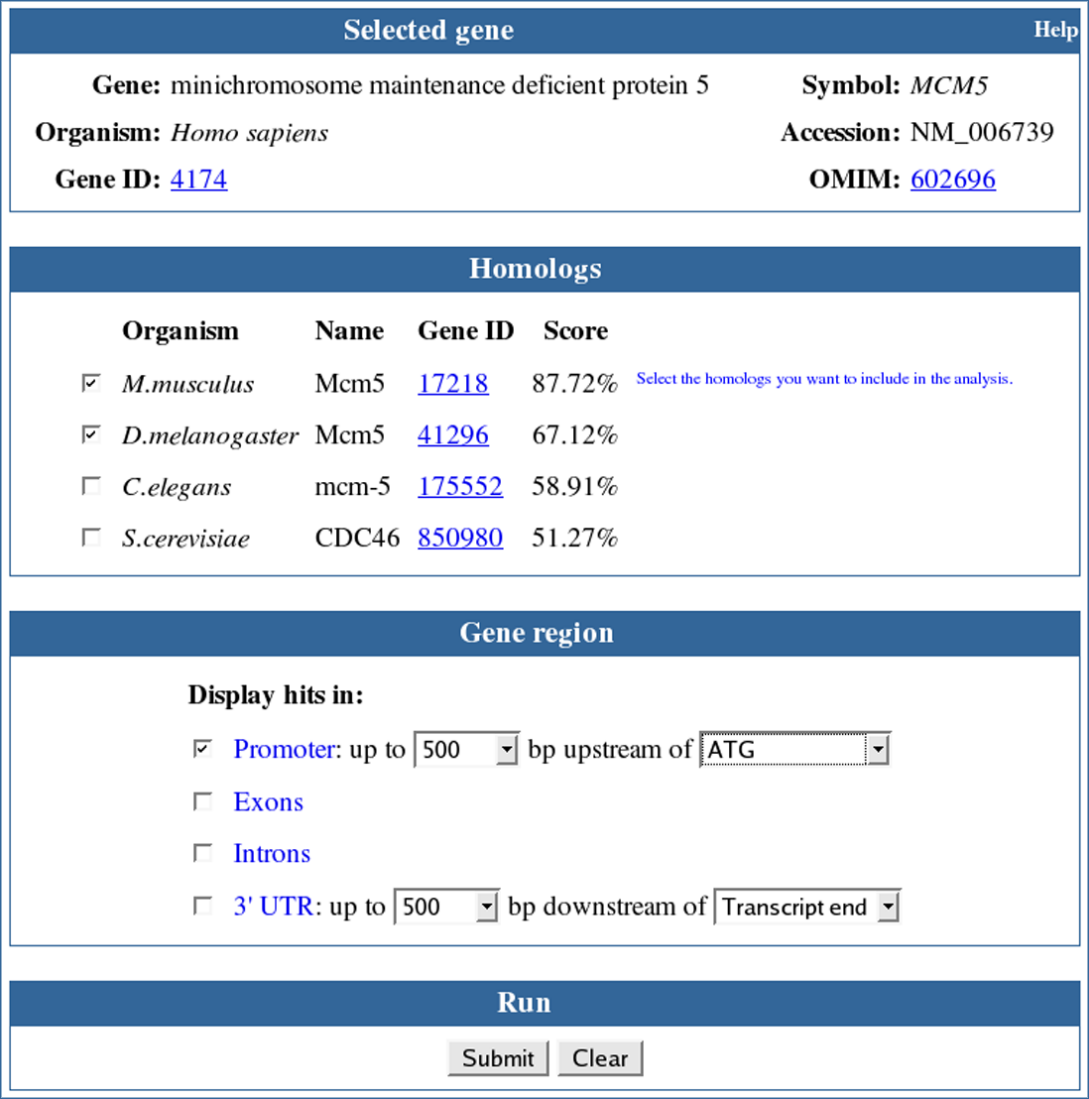
**The selection page of the search engine**. The selection page for the *MCM5 *gene displays detailed information on the gene and its homologs available in our database, and allows the user to select the gene region to be scanned. The same region will be scanned for all homologs included in the search.

We designed and implemented a web-based application, called MAPPER (Multi-genome Analysis of Positions and Patterns of Elements of Regulation), to facilitate the retrieval of putative TFBSs in a given sequence based on the library of 1,079 HMM models described above. The interface takes as input a gene identifier (e.g. NCBI Gene ID, RNA accession number) or a user supplied sequence in FastA format. The user then selects the models to be used (all, TRANSFAC or JASPAR only) and has the option to build his/her own model starting with a multiple sequence alignment of binding sites in FastA format. The search can be performed on the entire gene region flanked by a user-specified distance upstream and downstream, on a specified gene region (promoter, introns, exons, 3'-UTR) or within a certain distance upstream of the ATG or the start of the transcript (Figure 2). If a gene identifier is provided the program will also display the actual nucleotide sequence scanned (in FastA and Genbank format), a useful option in the case of those genes for which discrepancies exist between different annotations.

**Figure 3:**
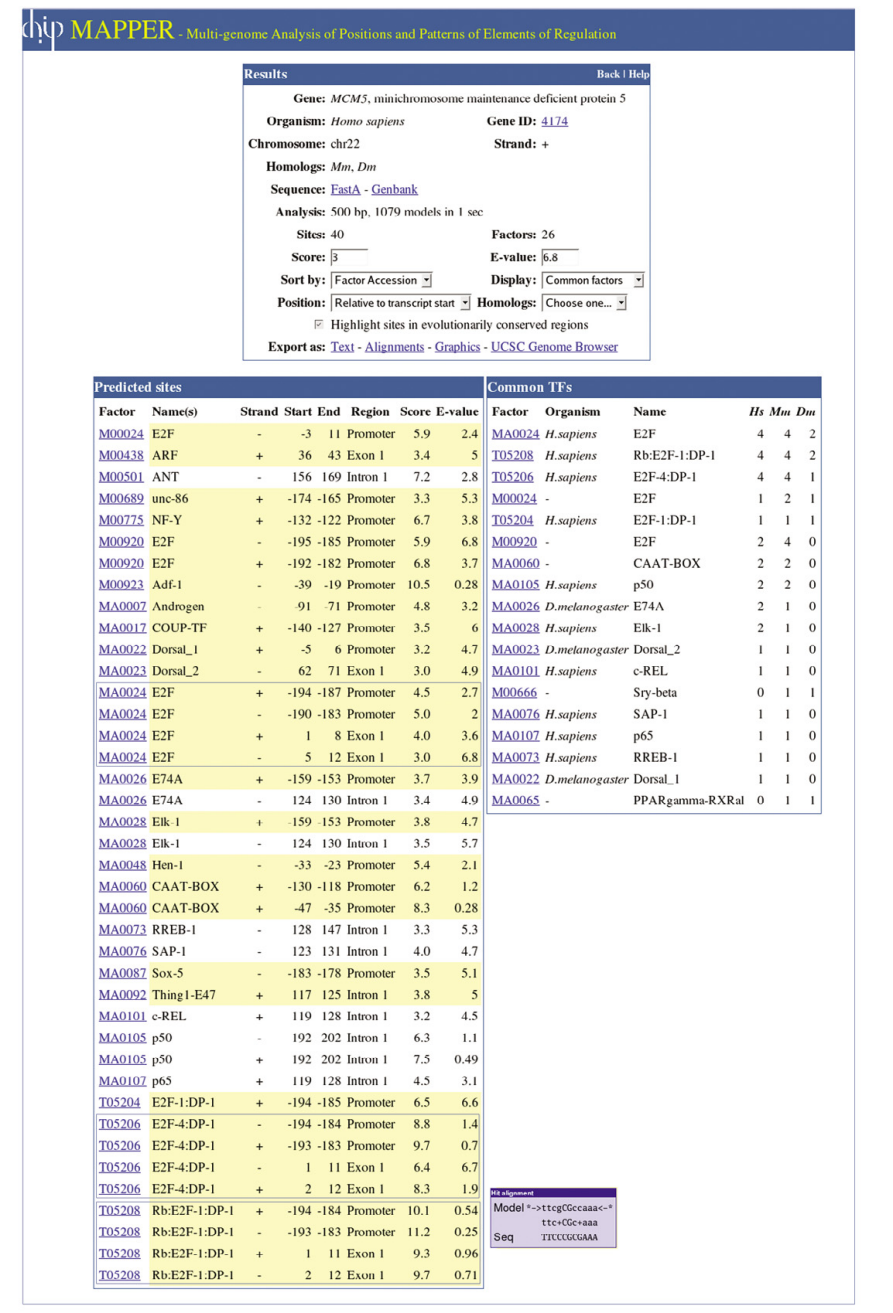
**The output of the query for the human *MCM5* gene**. The output was edited to highlight the E2F binding sites discussed in the text. The hit alignment window shows the match between the sequence at positions +2 to +12 from the transcript start and model T05206. The set of hits can be sorted by position, name or accession number of the factor. The position of the hits can be displayed with respect to the start of the transcript, the ATG or as absolute coordinates on the chromosome. The page can display the list of common factors that bind to the same selected region in the homologs included in the analysis, the factors on the list that are known to physically interact or the different classes to which they belong. In addition, the hits occurring in evolutionarily conserved regions can be highlighted.

**Figure 4:**
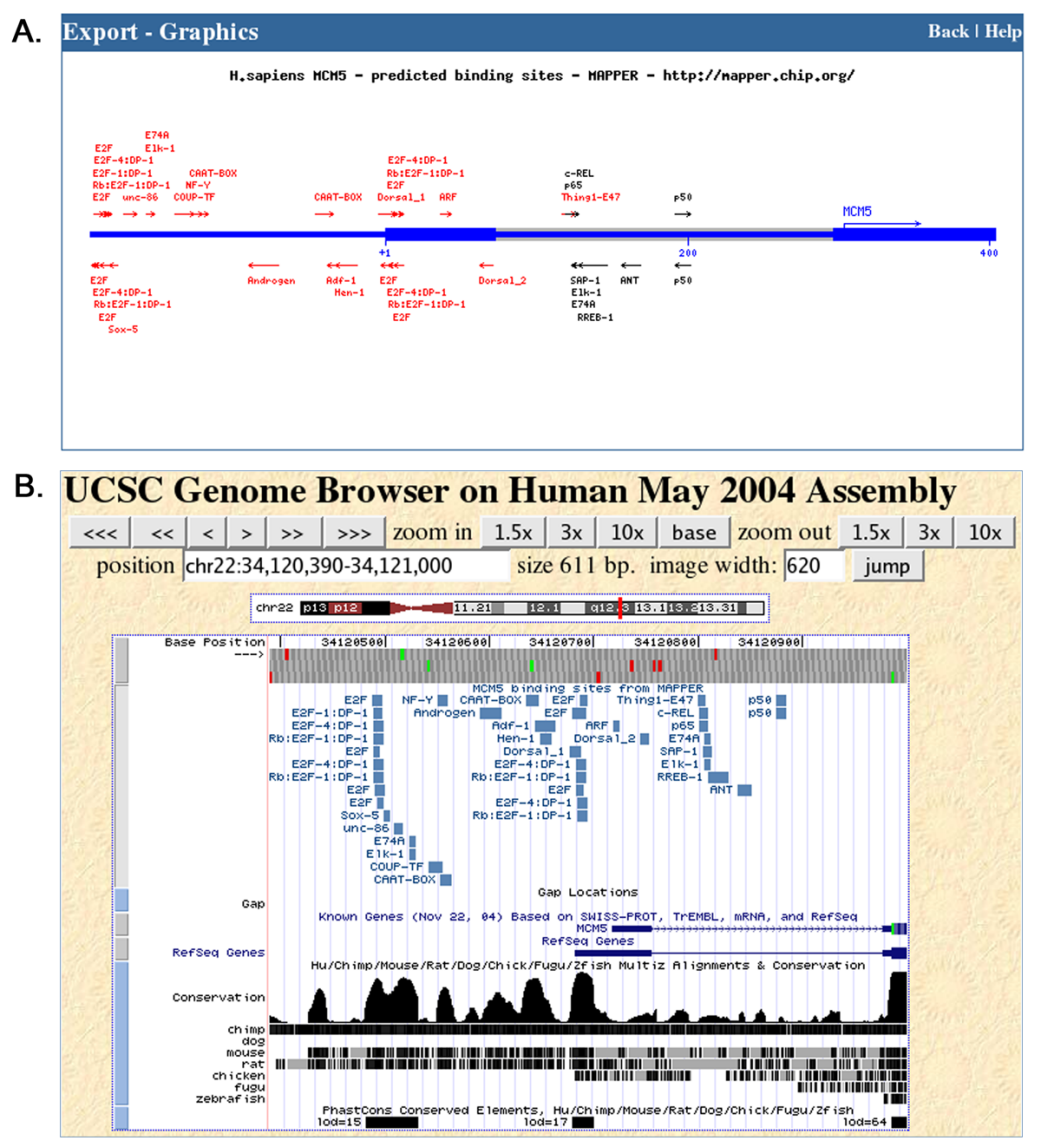
**Different representations of the set of putative TFBSs in the human *MCM5 *gene promoter**. A. Graphical representation of the hit set presented in Figure 3. B. The hit set was exported to the UCSC Human Genome Browser as a custom track. The region displayed in this image extends to 500 bp upstream of the coding sequence start. Note that the clusters of predicted binding sites correspond to peaks in the human/mouse conservation track at the bottom, suggesting that those regions are functional. The positions of the most conserved elements displayed in the conservation track are the ones used in the previous page to highlight hits in evolutionary conserved regions (see Methods for details).

The user can choose to display all hits for a given sequence, or only the hits for factors that are common across the orthologs of that sequence (if present in the HomoloGene database). The output of the system is the list of putative hits found in the specified conditions – default score and *E*-value thresholds are 0 and 10 respectively (Figure 3). For each hit the system displays the model used to retrieve it, its location, score and *E*-value and (in a pop-up window) the alignment between the model and the sequence at that site. The hit set can be sorted by position (from the ATG or the start of the transcript for genes supplied via an identifier, or from the beginning of the sequence for FastA sequences), by model name, model accession, score or *E*-value. In addition the results page highlights adjacent sites (situated within 50 bp from each other) retrieved for TFs that are known to physically interact with each other (as annotated in the TRANSFAC database), and also the classes of TFs for which putative sites were found. For each TRANSFAC factor-derived or JASPAR-derived model the organism in which the factor was described is specified. The user can choose to highlight hits in evolutionarily conserved regions representing the most conserved elements between sets of organisms provided by the UCSC Genome Browser annotations (see Methods for details). Figure 4A shows a graphical representation of the position and orientation of the hits listed in Figure 3. Arrows are drawn to scale and in some cases represent the sum of overlapping sites for the different transcription factors that are listed above or beneath them. Hits occurring in evolutionarily conserved regions are displayed in red. While the results page in Figure 3 lists and sorts all putative TFBSs independently from each other, the graphical representation in Figure 4A makes it easy to identify regions in which more than one model (corresponding to the same factor or to closely related factors) detects a binding site making it, therefore, more likely to represent a true binding site. The set of hits can also be exported and displayed in the UCSC Genome Browser using its "custom tracks" feature (Figure 4B). This allows the user to view the TFBSs in the general context of the genomic region in which they appear and to take advantage of the powerful visualization tools of the UCSC Genome Browser in order to highlight important features of the genomic region.

**Figure 5:**
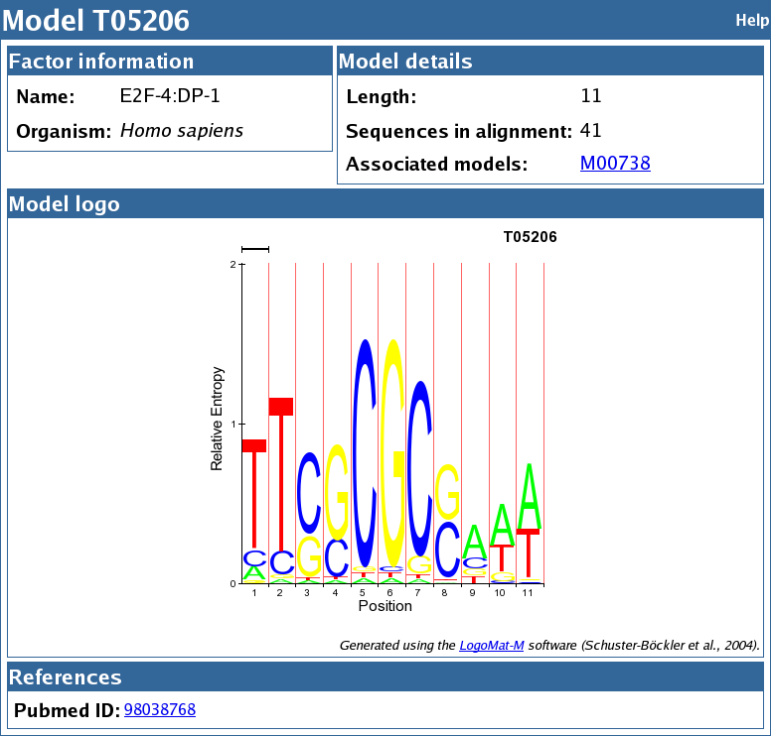
**The page for model T05206 for E2F-4:DP-1**. The model page displays detailed information regarding the model including the name and (if available) organism and classification of the factor, the model length, the number of sequences in the alignment used to train the model and the references used to select these sequences. The page also displays the HMM logo generated using the LogoMat-M software [89].

Figures 3 and 4 present the output of MAPPER when the human *MCM5 *gene (Entrez Gene ID 4174) is used as an example. The promoter of the human *MCM5 *gene contains multiple experimentally characterized binding sites for the E2F transcription factor. These binding sites were retrieved by our search, and were found to be conserved across the human, mouse and *Drosophila MCM5 *orthologs. *MCM5 *genes code for proteins involved in the initiation of DNA replication [83], and are members of the MCM family of chromatin-binding proteins that participate in cell cycle regulation. The E2F family of transcription factors plays a critical role in the control of cell proliferation and consists of six factors, E2F-1 to E2F-6, that heterodimerize with two other subunits, DP-1 and DP-2; the activity of these complexes is modulated by the retinoblastoma tumor suppressor protein (pRB) that binds E2F [84]. TRANSFAC and our database contain multiple models describing the binding sites in target genes characterized for different combinations of E2F and DP proteins, complexed or not with pRB. Below, we refer to these models generically as "E2F" models given the fact that, while the transcriptional role of E2F family members is different given the identity of the E2F and DP moieties that forms the complex [85], no specificity has been detected *in vivo *for the association of particular complexes to known E2F-regulated promoters [86,87]. Experimental evidence showed that the upregulation of the human *MCM5 *gene in response to growth stimulation is mediated by the binding of E2F to four sites within the *MCM5 *promoter, and that mutations in these sites abolish this response [88]. The four E2F binding sites consist of two sets of overlapping sequences running on opposite strands and were mapped by RNase protection assays to positions -194 to -183 and +2 to +13 respectively, relative to the start of the transcript [88]. In our search, three models for the E2F family (MA0024 for E2F, T05206 for E2F-4:DP-1 and T05208 for Rb:E2F-1:DP-1) retrieved all four E2F sites at the location and in orientation described in the literature (Figures 3 and 4). To simplify the display in these figures and to highlight the four E2F binding sites retrieved by the three models, a more stringent set of parameters was used for the query (500 bp upstream of the ATG, score > 3, *E*-value < 6.8). Figure 3 shows the list of all TFBSs retrieved given these input parameters with the four E2F binding sites described by Ohtani et al. boxed, as well as the list of factors for which putative binding sites where found also in the other two *MCM5 *homologs selected (mouse and *Drosophila MCM5*). For each hit in the listing the model identifier is displayed as a double link, to a pop-up window showing the match between the sequence and the model (Figure 3) and to a separate page giving detailed information regarding the model including its length, the number of sequences in the training set, associated models, HMM logo [89], and the references used to build the alignment (Figure 5).

## Discussion

Our method offers several advantages over other similar tools, with respect to the extent and quality of the models it uses, its sensitivity and specificity, and the overall functionality of the web-based interface.

MAPPER includes a large database of profile HMMs corresponding to 888 TF entries that was built using the data provided by the TRANSFAC and JASPAR databases, consisting of sets of experimentally validated binding sites for several hundred TFs. In addition to the models based on optimal alignments provided by TRANSFAC and JASPAR, our database includes a large number of additional models generated by extracting the representative motif from the "raw" binding site sequences contained in TRANSFAC with the program MEME (Multiple Expectation-maximization for Motif Elicitation) [90] that usually provide a tighter definition of the binding site specificity. As a result our method can make use of a larger number of models that provides an increased ability to detect putative binding sites. In many cases, several models are available for a single TF; in addition to increasing the probability of detecting a binding site for the factor, this redundancy also allows the user to evaluate whether a putative site is a "true" one (if it is detected by multiple models) or a potential false positive. Although the Plan7 architecture on which HMMER is built does not take into account the dependencies between the nucleotides within a site and, similarly to NWMs, weights each state independently [74], several features of the HMMER modeling and search procedure confer an added level of generality to HMMs as compared to NWMs built upon the same alignments. First, profile HMMs model insertions, deletions and allow fragment matches to the model [74]. This property becomes significant in the case of those TFs that bind to sites comprised of half sites separated by spacer regions of variable length (as for example nuclear receptors); while insertion and deletions are rare in the functional half sites they can occur with higher frequency in the spacer regions that are much more divergent [91]. Moreover, allowing fragment matches to the model ensures that binding sites that may contain a well defined half-site and an imperfect one or half-sites separated by long spacers can still be retrieved as fragment matches to the model. Secondly, all hits returned by HMMER are subject to a bias composition filtering based on a second null model that is computed for each alignment and leads to a rescoring of the hits penalizing the ones for which the nucleotide composition is biased [74]. Even in equal performance conditions, as could be the case for short alignments that do not allow insertions or deletions or fragment matches to the model, this filtering alone would still confer an advantage to using HMMER versus NWMs.

Using profile HMM for modeling bindings sites has also limitations. To build a model HMMER converts the observed counts in the training set into probabilities by combining the actual counts with pseudocounts from priors, in this case single-component Dirichlet priors [74]. The latter can have a more pronounced effect and can bias the model in the case in which the number of sequences in the training set is low. These cases would be difficult to model accurately by any statistical approach (including NWMs) and their suitability for the desired analysis will have to be evaluated case by case by the user. To facilitate this, we report for each model the length, the number of sequences in the training set, the HMM consensus (for matrix-derived models), the HMM logo [89], the other associated MAPPER models, and the references used to curate the binding sites used in the training set. The presence of models trained on small number of sequences usually does not represent a problem, as in the large majority of cases MAPPER makes available multiple models for any given TF.

We compared the predictive performance of our method with that of several other similar computational tools, by testing them on a dataset of over 100 experimentally determined binding sites as well as on synthetic data. The factors for which experimentally characterized sites were tested were selected so that they bind sites with different overall organization and belong to different categories of TFs such as fork head TFs (E2F and HNF-3), MADS box (MEF-2), helix-turn-helix/homeo domain (HNF-1), Cys4 Zn finger of nuclear receptor type (ER, GR and HNF-4), beta-scaffold factors with minor groove contacts (p53), and IPT/TIG domain (Su(H)). As presented in the results section our method correctly identified 15 out of 17 binding sites reported in the literature for p53, Su(H) and MEF-2. Moreover, from a collection of 89 binding sites for six other TFs (E2F, ER, GR, HNF-1, HNF-3 and HNF-4) our method identified 98% to 100% of the true positives with false positive ratios ranging from 0% to 16% (or 24% when a non-optimal model was used). The other methods tested (Match, Patser, LMM and ScanACE) either retrieved a comparable number of true positives at the expense of higher false positive ratios (as for example Match and Patser) or attained lower false positive ratios at the expense of missing a high number of true positives (as for example ScanACE and, in some cases, LMM).

Although encouraging, the results of this evaluation cannot be easily extrapolated to a scenario in which very long sequences (up to an entire genome) are scanned with hundreds of models. In order to make our analysis more general, we performed a large-scale evaluation using synthetic data for 491 models in our database (46% of the total number) for which a HMM and a NWM was available and compared the performance of our method with the one of Match in scanning sequences of 50 kb in length. Models that performed very poorly for one method or the other or both were filtered out and among the remaining 386 models 74% showed a statistically significant difference based on a Bonferroni corrected Wilcoxon signed rank test. Among the latter our method performed better in 96% of the cases.

However, we recognize that the entire enterprise of TF binding site annotation is burdened by the challenge of a robust definition of what constitutes a true positive, even those based on binding studies. For example, Tronche [92]and others note the evolutionary conservation of binding sites for genes transcribed in tissues that do not even express the transcription factor. In these instances as in others, computational or even biochemical binding assays are only a first step on the path to focused functional studies.

Finally, the MAPPER interface offers several advantages over other similar tools. It accepts as input a user-supplied FastaA sequence or a gene identifier for any annotated gene in the human, mouse, fly, worm or yeast genomes. It can use in the search all or each of the different categories of models in our database (based on TRANSFAC matrices, TRANSFAC factors or JASPAR matrices) or a model built on a multiple-sequence alignment supplied by the user. The results are presented in a simple yet comprehensive manner providing detailed information regarding the gene, the sequence scanned and the putative sites retrieved, and powerful graphical and export options facilitate the analysis and the interpretation of the results.

## Conclusion

The purpose of our work was to establish a methodology for the detection of TFBSs in multiple genomes endowed with enough sensitivity and specificity to be effective in large-scale analysis (such as generating a whole-genome map of binding sites for a collection of TFs). Accomplishing this requires a large library of high-quality TFBS models and a computational method able to reliably detect instances of the models in a given DNA sequence.

The model library used by our program was created from the data contained in the TRANSFAC and JASPAR databases, with a procedure that generated over a thousand high-quality models. The computational method we implemented relies on HMM profiles built from nucleotide sequence alignments. Using HMM profiles instead of NWMs is a powerful way for capturing the characteristics of a binding site and several observations suggest that our method is reliable and performs well. First, HMM profiles for selected factors retrieved binding sites in the promoter regions of genes used as controls with high specificity, as described in the Results section. Secondly, on an extended collection of experimentally characterized TFBSs, our method identified 98% to 100% of the true positives with a false positive ratio that was consistently smaller than the ones reported by the other methods tested. Thirdly, ROC and True Positive Proportion tests performed on a large number of models for which both a NWM and a HMM was available showed that in the majority of the cases our method performs significantly better than a NWM-based program such as Match. This translates into an increased ability to detect true binding sites while reducing the number of false positive sites reported. Finally, our method takes advantage of a larger set of models for a given TF, and this results in an increased ability to detect true hits.

The web-based interface was design to maximize usability and to facilitate the analysis of the retrieved hits; it has simple and flexible input requirements, a clear and comprehensive display of the results and powerful graphical and export options.

The current work and its future extensions make available a novel and reliable method for the identification of TFBSs that, used in combination with existing molecular genetic information and biological validation, represents a powerful tool for understanding the logic of combinatorial regulation. The search engine can be seen as the foundation for more advanced applications, such as highlighting patterns of TFBSs involved in the regulation of particular genes, assessing the conservation of such patterns across multiple genomes, or identifying the TFBSs overrepresented in a set of coexpressed genes.

## Methods

### Genomic sequences, homology and conservation information

Genomic sequences and annotations were downloaded from the UCSC Genome Bioinformatics site [93,94] and correspond to the following releases: *Homo sapiens *– hg17, *Mus musculus *– mm5, *Drosophila melanogaster *– dm1, *Caenorhabditis elegans *– ce2 and *Saccharomyces cerevisiae *– sg1. Homology information was obtained from the HomoloGene database Build 38.1 [95], containing clusters of genes that share a consistent ortholog relationship across three or more organisms [96]. Evolutionary conservation information was obtained from the UCSC Genome Browser [97] and consisted in the location and scores of the most conserved elements identified using the phastCons program [98] between the following sets of organisms respectively: human, chimp, mouse, rat, dog, chick, fugu and zebrafish; *Drosophila melanogaster*, *D. yakuba*, *D. pseudoobscura *and *A. gambiae*; *Caenorhabditis elegans *and *C. briggsae*; *Saccharomyces cerevisiae*, *S. paradoxus*, *S. mikatae*, *S. kudriavzevii*, *S. bayanus*, *S. castelli *and *S. kluyveri*. The option "highlight hits in evolutionarily conserved regions" of the results page of the interface emphasizes hits that fall in or within a distance of 100 bp upstream to 100 bp downstream of these elements for each appropriate genome.

### Generating the multiple sequence alignments of binding sites

The flat files of TRANSFAC Professional version 8.1 were parsed to extract two types of alignments: nucleotide sequences used to generate the TRANSFAC NWMs and the nucleotide sequences referenced in the description of the TRANSFAC factors (see below). The vast majority of matrix entries in TRANSFAC lists the accession numbers of the factor(s) associated with that matrix (multiple factors are usually orthologs from different organisms) and the accession numbers of the nucleotide sequences used to generate the matrix referred to below as "sites". Moreover, for each factor TRANSFAC lists which organism the factor belongs to and the accession numbers of the sites described for the factor in target genes. One factor can be linked with more than one matrix, and more than one matrix can describe the same factor. Not all matrices have associated site identifiers, and, more importantly, not all factors that have associated sites were used to build NWMs. Therefore, to extract the maximum amount of information, the TRANSFAC files were parsed following not only the links from "matrices" to "sites" but also the links from "matrices" to "factors" and from there to "sites". We called the alignments retrieved following the links from "matrices" to "sites" matrix-derived alignments. These were optimal multiple sequence alignments that were used as such to build HMMs called matrix-derived models and having accession numbers starting with "M". Nucleotide sequences retrieved following the links from "matrices" to "factors" and from there to "sites" were first processed in order to extract the underlying motif using the MEME program [90] downloaded from [99]. For each set of sequences, the MEME search was conducted separately on the forward and on the forward and reverse strands and the best motif was selected taking into account its length and *E*-value; this selection was also verified by manual curation. The resulting MEME alignments, called factor-derived alignments, were used to build HMMs called factor-derived models that have accession numbers starting with "T".

Motifs extracted using Gibbs sampling from nucleotide sequences of binding sites and used to build the JASPAR matrices were extracted by parsing the matrix site files downloaded from [100]. The resulting alignments, called JASPAR-derived alignments, were checked against the Jaspar matrices and used to build HMMs called JASPAR-derived models designated with accession numbers starting with "MA". The accession numbers of the HMM models are the same as the corresponding entries in the TRANSFAC and JASPAR databases. To estimate the number of TFs that have corresponding models in MAPPER, we counted them as distinct if they had different names, although in several cases in TRANSFAC and JASPAR entries with different names may refer to the same TF or TF family, or slightly different names may refer to isoforms of the same TF.

### Generating HMMs from alignments using HMMER

Profile Hidden Markov models were generated using the HMMER package (version 2.2 August 2001) available at [74]. The null model used to generate the models employed equal probabilities for all four nucleotides and took into account the fact that TFBSs can occur frequently throughout the sequence scanned. Therefore we used in the null model a p1 value for the *G*→*G *transition controlling the expected length of the target sequences [74] equal to 0.98 instead of the default value of 0.999, thus assuming that two sites for the same TF may occur 50 bp and not 1000 bp apart as in the default model. This significantly decreased the likelihood of retrieving true positive hits with negative scores (S.R. Eddy, personal communication).

The HMMER function *hmmpfam *searches a sequence or a database of sequences against a library of HMM models, and characterizes each hit it returns by two parameters: the score and the *E*-value. The score is the logarithm in base 2 of the ratio *P*(seq|HMM)/*P*(seq|null), where *P*(seq|HMM) is the probability of the target sequence according to the HMM model and *P*(seq|null) is the probability of the sequence according to a null model distribution. The greater the score the better the match between the hit and the model is. The *E*-value, computed with respect to the number of the sequences in the database queried, is a measure of the expected number of false positives that will have scores equal to or larger than the score of the hit. The smaller the *E*-value, the more significant the hit is.

### HMMER control runs

First, a qualitative evaluation of the performance of the method was carried out by searching promoter sequences of selected genes that contain well characterized binding sites for specific TFs against the HMMs built for these factors. The factors selected as controls were mouse p53, human MEF-2 and *Drosophila *Su(H) – Suppressor of Hairless for which the alignment files M00761, T00505 and M00234 respectively, were used to construct and calibrate HMMs. For the promoter sequences of the genes used as controls the nucleotide positions and sequences of the characterized binding sites for these TFs were available in the literature and were compared with the ones of the hits returned by HMMER.

### Small-scale evaluation

As a starting point for this analysis, six datasets were prepared containing a total of 110 experimentally characterized binding sites for the following transcription factors: E2F, the estrogen receptor (ER), the glucocorticoid receptor (GR), the hepatocyte nuclear factors HNF-1, HNF-3 and HNF-4. The sequences for 27 E2F and 25 ER binding sites, located between well defined positions in the center of a 50 bp sequence containing flanking regions from the corresponding genes [78], were downloaded from [101]. For consistency and to facilitate the analysis of the results the remaining datasets were processed and written in the same format. The sequences of 16 GR binding sites were downloaded from [79,102]. 19 bindings sites for HNF-1, 11 for HNF-3 and 12 for HNF-4 [80] were obtained by parsing the datafiles at [103]. For these datasets the sequence of the sites listed in the Gibbs sampling log files were matched to the original fasta sequences and the flanking nucleotides extracted in a sequence of 50 bp total.

The methods used for comparison were accessed as follows: the Match code was supplied with the TRANSFAC professional 8.2 suite [19], Patser [9] was used at [104], LMM [67] was downloaded from [105], and ScanACE [6] was downloaded from [106]. For Match a value of 0.7 was used for both the matrix and the core similarity thresholds. Patser, ScanACE and the *hmmpfam *function of HMMER were used with default parameters. LMM was used with a window size of 15 and default values for the other parameters.

For each dataset we used the NWM provided by TRANSFAC as input for the NWM-methods and its corresponding HMM model as input for HMMER. An alternative, better performing HMM model for the factor (designated with accession numbers starting with "T") was always included for HMMER. In three cases, for LMM that contains only the publicly available TRANSFAC matrices we had to use the closest available matrix for the factor as an input. The following matrices and corresponding HMM models were used for this analysis: for E2F V$E2F_02 (equivalent to M00050) and T05206; for ER V$ER_Q6_02 (equivalent to M00775), T00258 and V$ER_Q6 (for LMM); for GR V$GR_Q6_01 (equivalent to M00921), T05076 and V$GR_Q6 (for LMM); for HNF-1 V$HNF1_Q6 (equivalent to M00790), T01211 and V$HNF1_01 (for LMM); for HNF-3 V$HNF3ALPHA_Q (equivalent to M00724) and T00371; for HNF-4 V$HNF4ALPHA_Q6 (equivalent to M00638) and T00372. For each factor the binding sites included in the test datasets were checked against the ones in the training set on which the corresponding matrix and HMMs were built. 21 sites that were in common were eliminated from the test set resulting in a filtered dataset of 89 binding sites that can be downloaded following the appropriate link from Additional File 1. No information regarding the exact sites used to build the alternative LMM matrices was available in TRANSFAC so these matrices may very well overfit the test set.

The output of each method was parsed to identify the true and the false positives among the hits retrieved. Hits retrieved on both strands and overlapping more than 75% were counted as one hit. True positives were defined as hits that are either contained in or overlap at least 60% of the sequence of the known binding site; all other hits were considered false positives. For each run the number of distinct sequences containing at least one true positive was reported. Hits were sorted by score and the percent of false positives was calculated as the ratio between the false positives and the sum of false positives and true positives that have to be retrieved until at least one true positive was found for each of *n *sequences from the dataset. The value of *n *was chosen for each dataset based on the following rule: if in four cases corresponding to three different methods (the two HMMER cases, Match and Patser) the maximum number of unique sequences was identified, we used this value as a cutoff. Otherwise we used the value immediately below the maximum number. This percentage was not computed for methods that missed more than 2 sequences from the dataset.

### Large-scale evaluation

We compared the sensitivity and specificity of our method against those of a NWM-based method (Match) by carrying out a large-scale analysis of their performance on synthetic data using 491 models in our database corresponding to the TRANSFAC and JASPAR matrices for which both a NWM and a HMM, built from the same multiple sequence alignment, were available. For each of these models we generated a 50,000 bp random sequence and we planted 100 "synthetic" binding sites into it, at random locations [81]. Before a hit was planted the random sequence was scanned to eliminate any potential matches that could occur by chance. The algorithm used to generate the synthetic binding sites builds a simple Markov chain by reading the original multiple sequence alignment and represents each step in the chain as a 6-by-6 matrix of transition frequencies (the states include A, C, G, T, N and gap). Each sequence in the alignment is scanned sequentially, and the matrix element corresponding to each transition is incremented. In the end, the counts are converted into probabilities by normalizing them to 1. The Markov chain was used to generate new sequences by choosing a starting base at random according to the marginal probabilities of the bases in the first position, and by selecting at random a transition from each successive matrix at each step. This procedure prevents transitions that never appear in the alignment from being generated, as could instead happen if the synthetic sites had been generated by sampling from the probability distribution described by the NWM, while at the same time preserving the "core" sequence (the most conserved nucleotides) that Match relies on. Therefore, this method is well suited for generating sequences that can be recognized by both HMMER and Match, without giving an unfair disadvantage to any of the two methods. This method was favored over inserting at random binding sites from the alignments used to generate the NWM or HMM in order to keep the training and test set separate and to evaluate the two methods based on their ability to detect sequences that are similar but not identical with the one already reported as it would be expected for novel *bona fide *binding sites. We scanned the resulting sequence with both HMMER (with a threshold on the E-value equal to 20) and Match (with both core matrix and similarity matrix thresholds equal to 0.7), and we compared the results of both programs against the known locations of the synthetic binding sites. The whole process was repeated 20 times, and the results were analyzed in two different ways, by using modified ROC curves [82] and True Positive Proportion (TPP) tests described below.

We generated ROC plots to obtain an indication of the overall performance of both methods. However, while our methodology provides a definition of true and false positives, it does not explicitly define the set of true negatives. Since both programs scan the nucleotide sequence assigning a score to every position in the sequence and moving one base at a time, the effective number of true negative hits should be the length of the sequence minus the number of planted TFBSs, that is, the number of positions that were tested and were not found to match the TFBS pattern. This obviously results in a heavy imbalance between the number of true positives and the number of false positives, making it hard to evaluate the ROC curves in the commonly used way. For example, the areas under the curves that are normally used as a measure of predictive performance will always be very close to 1 and very similar to each other. Therefore, following the method of Gribskov and Robinson [82] we used ROC_50 _curves, plotted until 50 false positives are found, and we computed the areas under them (ROC_50 _areas). Models for which one or both method attained average areas below 0.25 were filtered out as their comparison was not meaningful. To determine if the values of the two sets are statistically different we performed a Wilcoxon signed rank test at significance value *α *equal to 0.05 with a Bonferroni correction of *α *divided by the number of independent tests (491). The result of this test is presented in Table 2 of Additional file 1.

In addition to the ROC curves, we also used two alternative tests, assessing the True Positive Proportion of hits retrieved by each method. For each sequence, we sorted the list of predicted hits by score, from highest to lowest, and we determined the percentage of true hits retrieved by each method within the first *n *reported hits and the amount of false positive hits (expressed as percentage) that one needs to accept in order to identify the first *m *true positive hits. The values used for *n *and *m *were 30, 50, 70 and 90. One method was considered to outperform the other in the TPP tests if it scored strictly better (showed a higher or lower percentage, depending on the test) for three out of the four *n *or *m *values and equally or better in the remaining one. The complete results of these tests are reproduced in Tables 3 and 4 of Additional File 1.

### Database construction, website development and software environment

Genomic annotations from the UCSC Genome Browser, TRANSFAC, JASPAR and HomoloGene information were used to build a MySQL relational database storing data about genes, transcription factors, and their binding sites.  We implemented a web-based system, accessible at , that allows users to search for the putative TFBSs in any region of a gene and of its orthologs, or in an arbitrary user-supplied sequence.  This resource also offers access to a previously described database of pre-computed TFBSs found in the upstream sequences of all genes in the human, mouse and *Drosophila* genomes [107], that was generated using a methodology similar to the one described in this paper.  The application is written in Common Lisp and relies on a development environment for web-based applications developed by the authors.  

## Abbreviations

HMM – hidden Markov model; NWM – nucleotide weight matrix; ROC curve – receiver operating characteristic curve; TF – transcription factor; TFBS – transcription factor binding site; TPP test – true positive proportion test.

## Supplementary Material

Additional File 1 Word file containing links to the factors table and the results of the small-scale and large-scale evaluations. Click here for file

Additional File 2 Excel file containing detailed results of the small-scale evaluation. Click here for file

## Figures and Tables

**Table 1:** Results of the small-scale evaluation.

**Factor**	**Sites**	**Method**	**Model**	**Distinct sequences identified**	**Target**	**True positives**	**False positives**	**False positive ratio**
**E2F**	27	HMMER	M00050	27	27	36	8	18.20%
			T05206	27	27	27	4	12.90%
		Match	V$E2F_02	27	27	36	7	16.30%
		Patser	V$E2F_02	27	27	36	8	18.20%
		LMM	V$E2F_02	18	27	18	3	n/a
		ScanACE	M00050	12	27	12	3	n/a
**ER**	17	HMMER	M00959	16	16	22	7	24.10%
			T00258	17	16	16	2	11.10%
		Match	V$ER_Q6_02	17	16	24	7	22.60%
		Patser	V$ER_Q6_02	17	16	24	8	25.00%
		*LMM*	*V$ER_Q6*	*15*	*16*	*15*	*0*	*0.00%*
		ScanACE	M00959	8	16	11	1	n/a
**GR**	7	HMMER	M00921	7	7	10	2	16.70%
			T05076	7	7	7	1	12.50%
		Match	V$GR_Q6_01	7	7	9	3	25.00%
		Patser	V$GR_Q6_01	7	7	9	7	43.70%
		*LMM*	*V$GR_Q6*	*6*	*7*	*6*	*1*	*14.30%*
		ScanACE	M00921	4	7	4	1	n/a
**HNF-1**	18	HMMER	M00790	18	18	19	0	0.00%
			T01211	18	18	18	0	0.00%
		Match	V$HNF1_Q6	18	18	22	3	12.00%
		Patser	V$HNF1_Q6	18	18	29	1	3.30%
		*LMM*	*V$HNF1_01*	*16*	*18*	*16*	*0*	*0.00%*
		ScanACE	M00790	11	18	11	0	n/a
**HNF-3**	10	HMMER	M00724	10	10	10	1	9.10%
			T01049	10	10	10	2	16.70%
		Match	V$HNF3ALPHA_Q6	10	10	12	4	25.00%
		Patser	V$HNF3ALPHA_Q6	10	10	10	1	9.10%
		LMM	V$HNF3ALPHA_Q6	9	10	9	4	30.80%
		ScanACE	M00724	8	10	8	0	0.00%
**HNF-4**	10	HMMER	M00638	9	9	9	2	18.20%
			T00372	10	9	9	0	0.00%
		Match	V$HNF4ALPHA_Q6	9	9	9	2	18.20%
		Patser	V$HNF4ALPHA_Q6	10	9	9	2	18.20%
		LMM	V$HNF4ALPHA_Q6	7	9	7	0	0.00%
		ScanACE	M00638	3	9	3	0	n/a

The "Sites" column contains the number of sequences containing experimentally validated binding sites provided as input. "Target" represents the number of binding sites to be retrieved by a method to be considered successful, and "Distinct sequences identified" is the number of distinct sequences in which at least one true positive was detected. Because of partially overlapping hits, the actual number of true positives reported may be higher than the true number of sites. Not all matrices tested were available in the LMM matrix library; for those cases, the results obtained using the closest available LMM matrix are displayed in italics.

**Table 2:** Comparative performance of the TFBS detection methods tested.

**Performance value**	**Method**					
	
	**HMMER M model**	**HMMER T model**	**Match**	**Patser**	**LMM**	**ScanACE**
% true positives identified	98%	100%	99%	100%	80%	52%
minimum false positive ratio	0.00%	0.00%	12.00%	3.30%	0.00%	0.00%
maximum false positive ratio	24.10%	16.70%	25.00%	43.70%	30.80%	0.00%

A summary of the results presented in Table 1. The first row displays the overall percentage of validated binding sites detected by the different methods. The next two rows display the minimum and maximum percentage of false positive hits found by each method across the six different datasets tested.
